# “Insights into vessel perforations during thrombectomy: Characteristics of a severe complication and the effect of thrombolysis”

**DOI:** 10.1177/23969873241272542

**Published:** 2024-08-22

**Authors:** Victor Schulze-Zachau, Nikki Rommers, Nikolaos Ntoulias, Alex Brehm, Nadja Krug, Ioannis Tsogkas, Matthias Mutke, Thilo Rusche, Amedeo Cervo, Claudia Rollo, Markus Möhlenbruch, Jessica Jesser, Kornelia Kreiser, Katharina Althaus, Manuel Requena, Marc Rodrigo-Gisbert, Tomas Dobrocky, Bettina L Serrallach, Christian H Nolte, Christoph Riegler, Jawed Nawabi, Errikos Maslias, Patrik Michel, Guillaume Saliou, Nathan Manning, Alexander McQuinn, Alon Taylor, Christoph J Maurer, Ansgar Berlis, Daniel PO Kaiser, Ani Cuberi, Manuel Moreu, Alfonso López-Frías, Carlos Pérez-García, Riitta Rautio, Ylikotila Pauli, Nicola Limbucci, Leonardo Renieri, Isabel Fragata, Tania Rodriguez-Ares, Jan S Kirschke, Julian Schwarting, Sami Al Kasab, Alejandro M Spiotta, Ahmad Abu Qdais, Adam A Dmytriw, Robert W Regenhardt, Aman B Patel, Vitor Mendes Pereira, Nicole M Cancelliere, Carsten Schmeel, Franziska Dorn, Malte Sauer, Grzegorz M Karwacki, Jane Khalife, Ajith J Thomas, Hamza A Shaikh, Christian Commodaro, Marco Pileggi, Roland Schwab, Flavio Bellante, Anne Dusart, Jeremy Hofmeister, Paolo Machi, Edgar A Samaniego, Diego J Ojeda, Robert M Starke, Ahmed Abdelsalam, Frans van den Bergh, Sylvie De Raedt, Maxim Bester, Fabian Flottmann, Daniel Weiss, Marius Kaschner, Peter T Kan, Gautam Edhayan, Michael R Levitt, Spencer L Raub, Mira Katan, Urs Fischer, Marios-Nikos Psychogios

**Affiliations:** 1 Department of Diagnostic & Interventional Neuroradiology, Radiology & Nuclear Medicine Clinic, University Hospital Basel, Basel, Switzerland; 2 Clinical Research Department, University Basel, Basel, Switzerland; 3 Department of Neuroradiology, Ospedale Niguarda Ca’ Granda, Milano, Italy; 4 Vascular & Interventional Neuroradiology Section, Minimal Invasive NeuroTherapy Center, Heidelberg University Hospital, Heidelberg, Germany; 5 Radiology and Neuroradiology Clinic, University and Rehabilitation Clinic Ulm, Ulm, Germany; 6 Neurology Clinic, University Hospital Ulm, Ulm, Germany; 7 Interventional Neuroradiology, Hospital Universitari Vall d’Hebron, Barcelona, Spain; 8 Neurology Department, Hospital Universitari Vall d’Hebron, Barcelona, Spain; 9 Institute of Diagnostic and Interventional Neuroradiology, Inselspital, Bern University Hospital and University of Bern, Bern, Switzerland; 10 Department of Neurology with Experimental Neurology, Charité Universitätsmedizin Berlin, Campus Benjamin Franklin, Berlin, Germany; 11 Center for Stroke Research Berlin (CSBand Berlin Institute of Healths (BIH), Charité Universitätsmedizin Berlin, Berlin, Germany; 12 Department of Neuroradiology, Charité - Universitätsmedizin Berlin, Campus Mitte, Humboldt-Universität zu Berlin, Freie Universität Berlin, Berlin, Germany; 13 Berlin Institute of Health (BIH), BIH Biomedical Innovation Academy, Berlin, Germany; 14 Stroke Center, Service of Neurology, Department of Clinical Neurosciences, Lausanne University Hospital (CHUV), University of Lausanne, Lausanne, Switzerland; 15 Interventional Neuroradiological Unit, Service of Diagnostic and Interventional Radiology, Department of Medical Radiology, Lausanne University Hospital (CHUV), University of Lausanne, Lausanne, Switzerland; 16 Department of Interventional Radiology, Liverpool Hospital, Sydney, Australia; 17 Neurointervention and Neurovascular Clinic, Prince of Wales Hospital, Sydney, Australia; 18 Department of Diagnostic and Interventional Neuroradiology, Augsburg University Hospital, Germany; 19 Department of Neuroradiology, University Hospital Carl Gustav Carus, Dresden, Germany; 20 Department of Radiology, University Hospital Carl Gustav Carus, Dresden, Germany; 21 Neurointerventional Unit, Radiology Department, Hospital Clínico Universitario San Carlos, Madrid, Spain; 22 Department of Interventional Radiology, Turku University Hospital, Finland; 23 Department of Neurovascular Intervention, Ospedale Careggi di Firenze, Florence, Italy; 24 NOVA Medical School, Lisbon, Portugal; 25 Department of Neuroradiology, Centro Hospitalar Universitário de Lisboa Central, Lisbon, Portugal; 26 Department of Diagnostic and Interventional Neuroradiology, Klinikum rechts der Isar, TUM School of Medicine, Technical University of Munich, Munich, Germany; 27 Department of Neurosurgery, Medical University of South Carolina, Charleston, SC, USA; 28 Department of Neurology, Medical University of South Carolina, Charleston, SC, USA; 29 Neuroendovascular Program, Massachusetts General Hospital & Brigham and Women’s Hospital, Harvard Medical School, Boston, MA, USA; 30 Neurovascular Centre, Departments of Medical Imaging & Neurosurgery, St. Michael’s Hospital, University of Toronto, Toronto, Canada; 31 Clinic for Neuroradiology, University Hospital Bonn, Bonn, Germany; 32 Department of Radiology and Nuclear Medicine, Luzerner Kantonsspital, Luzern, Switzerland; 33 Department of Neurology, Cooper Neurological Institute, Camden, NJ, USA; 34 Department of Neurosurgery, Cooper Neurological Institute, Camden, NJ, USA; 35 Department of Neurointerventional Surgery, Cooper Neurological Institute, Camden, NJ, USA; 36 Department of Diagnostic and Interventional Neuroradiology, Neurocenter of Southern Switzerland, EOC, Lugano, Switzerland; 37 University Clinic for Neuroradiology, Medical Faculty, Otto-Von-Guericke-University, Magdeburg, Germany; 38 Service de Neurologie, CHU de Charleroi, Charleroi, Belgium; 39 Service of Diagnostic and Interventional Neuroradiology, Geneva University Hospital, Geneva, Switzerland; 40 Department of Neurology, University of Iowa, College of Medicine, Iowa City, IA, USA; 41 Department of Neurosurgery, University of Iowa, College of Medicine, Iowa City, IA, USA; 42 University of Iowa, College of Medicine, Iowa City, IA, USA; 43 Miami Miller School of Medicine, Jackson Memorial Hospital, University of Miami Hospital, Miami, USA; 44 Department of Radiology, Universitair Ziekenhuis Brussel, Vrije Universiteit Brussel (VUB), Brussels, Belgium; 45 Department of Neurology, Universitair Ziekenhuis Brussel, Center for Neurosciences, Vrije Universiteit Brussel (VUB), Brussels, Belgium; 46 Department of Neuroradiology, University Hospital Eppendorf, Hamburg, Germany; 47 Department of Diagnostic and Interventional Radiology, University Düsseldorf, Düsseldorf, Germany; 48 Department of Neurosurgery, University of Texas Medical Branch, Galveston, TX, USA; 49 Department of Neurosurgery, University of Washington, Seattle, Washington, USA; 50 Neurology Clinic, University Hospital Basel, Basel, Switzerland

**Keywords:** Stroke, thrombectomy, thrombolysis, complication, intraoperative, intracranial hemorrhage

## Abstract

**Introduction:**

Thrombectomy complications remain poorly explored. This study aims to characterize periprocedural intracranial vessel perforation including the effect of thrombolysis on patient outcomes.

**Patients and methods:**

In this multicenter retrospective cohort study, consecutive patients with vessel perforation during thrombectomy between January 2015 and April 2023 were included. Vessel perforation was defined as active extravasation on digital subtraction angiography. The primary outcome was modified Rankin Scale (mRS) at 90 days. Factors associated with the primary outcome were assessed using proportional odds models.

**Results:**

459 patients with vessel perforation were included (mean age 72.5 ± 13.6 years, 59% female, 41% received thrombolysis). Mortality at 90 days was 51.9% and 16.3% of patients reached mRS 0–2 at 90 days. Thrombolysis was not associated with worse outcome at 90 days. Perforation of a large vessel (LV) as opposed to medium/distal vessel perforation was independently associated with worse outcome at 90 days (aOR 1.709, *p* = 0.04) and LV perforation was associated with poorer survival probability (HR 1.389, *p* = 0.021). Patients with active bleeding >20 min had worse survival probability, too (HR 1.797, *p* = 0.009). Thrombolysis was not associated with longer bleeding duration. Bleeding cessation was achieved faster by permanent vessel occlusion compared to temporary measures (median difference: 4 min, *p* < 0.001).

**Discussion and conclusion:**

Vessel perforation during thrombectomy is a severe and frequently fatal complication. This study does not suggest that thrombolysis significantly attributes to worse prognosis. Prompt cessation of active bleeding within 20 min is critical, emphasizing the need for interventionalists to be trained in complication management.

## Graphical abstract

**Graphical abstract:**
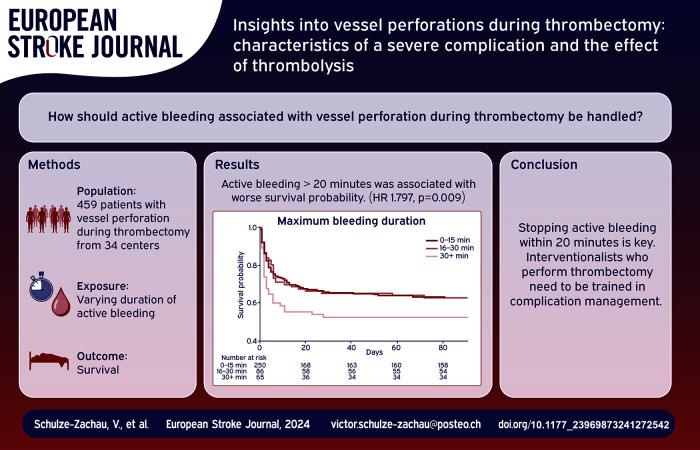

## Introduction

Since five randomized trials have shown overwhelming benefit for patients with acute ischemic stroke and large vessel occlusion (LVO) in 2015,^1^ the indications for thrombectomy have been continuously expanding.^2^ Patients presenting late after symptom onset or with unknown onset and patients with large infarcts are now largely considered candidates for thrombectomy.^3–9^ Furthermore, several studies are investigating whether thrombectomy may improve outcome in patients with medium/distal vessel occlusion (MDVO)^10^ or with mild neurologic deficit at presentation.^11^ In these patients, the volume of salvageable tissue is typically smaller and the natural course is less severe compared to LVO stroke.^10,12,13^ In addition, the natural course of the disease may be less predictable.^14^ Therefore, the treatment effect regarding functional outcome is assumed to be smaller compared to acute LVO stroke.^10^

Thrombectomy is an invasive treatment and contains risks. Periprocedural vessel perforation with intracranial hemorrhage is one of the most feared complications with a mortality of approximately 50%.^15–20^ Vessel perforation has been reported to be more frequent in MDVO compared to LVO thrombectomy,^17,18,21^ necessitating physicians involved in decision making for patients with MDVO to balance the risks and benefits of thrombectomy.^10^ But otherwise, literature on vessel perforations is scarce. However, if an invasive treatment is more and more considered for patients with smaller or less clearly predictable therapeutic yield, the potential risks need to be understood in-depth to (a) improve decision-making regarding treatment eligibility, (b) reduce the frequency of complications, and (c) optimize complication management.

The aim of our quality assurance project was (1) to characterize vessel perforation during thrombectomy including the management of active bleeding as well as the management of remaining arterial occlusions, (2) to compare perforation of large vessels (LV) to perforation of medium / distal vessels (MDV), and (3) to explore the effect of intravenous thrombolysis on clinical outcomes in patients with vessel perforation during thrombectomy.

## Patients and methods

Data of consecutive patients with vessel perforation during thrombectomy were retrospectively collected from multiple stroke centers.

### Patient selection

Patients were included if thrombectomy was performed between January 2015 and April 2023 and if intradural vessel perforation was confirmed by contrast agent extravasation during angiographic series. No other inclusion and exclusion criteria were applied. Collected data included patients’ baseline characteristics and details of the medical management and the neurovascular procedure. In a prior article,^18^ we reported on 277 patients also included in this study. The current study focuses on different aspects not discussed in the previous article: the effect of thrombolysis, the differentiation between LV perforation and MDV perforation and the importance of bleeding duration.

We differentiated the site of perforation in three categories: (1) LV perforation was defined as perforation of the intradural internal carotid artery, the M1 segment or the dominant M2 segment of the middle cerebral artery, the V4 segment of the vertebral artery or the basilar artery. (2) MDV perforation was defined as perforation of the non-dominant or co-dominant M2 segments or M3–M5 segments of the middle cerebral artery, A1–A5 segments of the anterior cerebral artery, P1–P4 segments of the posterior cerebral artery or perforation of the anterior or posterior communicating arteries. Perforation of vessels of similar diameter was considered MDV perforation. (3) Perforation of lenticulostriatal perforators, thalamic perforators or the anterior choroid artery was defined to be perforation of small vessels/perforator branches.

Modified Rankin Scale (mRS) at 90 days was collected as primary outcome measure and good functional outcome was defined as mRS 0–2.

Bleeding duration was derived from angiographic series: the time between the first and last series showing extravasation was defined as minimum bleeding duration and the time between the first series showing extravasation and the first series showing cessation of extravasation was defined as maximum bleeding duration. The bleeding duration was defined to be short (minimum bleeding duration 0–5 min and maximum bleeding duration 0–15 min), intermediate (6–20 min and 16–30 min) or long (> 20 min and > 30 min). These time intervals were chosen since preparation of material for hemostatic therapy was assumed to last 5–10 min and application of a first-line hemostatic strategy was assumed to take 15–20 min. Time from onset to admission was divided into three groups: (1) 0–6 h, (2) 6–24 h, and (3) other including unknown onset.

Ethical commission approval and patient consent were not required according to current local legislation as all data were anonymized before analysis and the project involved assessing safety and quality of routine patient management in the participating institutions.

### Statistics

Patient, occlusion, and perforation characteristics were described as mean with standard deviation for continuous variables and frequency with percentage for categorical variables.

We assessed effects of thrombolysis and potentially covariates on the primary outcome mRS at 90 days using ordinal shift analysis with proportional odds models: We fitted univariable models for pre-defined potential covariates. If a significant result was found in univariable models (*p* < 0.05), these variables were included in the final models. Covariates included were pre-stroke mRS, National Institutes of Health Stroke Scale (NIHSS) at admission, successful recanalization defined as Modified treatment in cerebral infarction score (mTICI) ⩾ 2b and perforation site (LV vs MDV). We adjusted for clustering of data by center by adding center as a random intercept to all models.

The association between bleeding duration (outcome) and different predictor variables was assessed using linear mixed models, accounting for clustering of data by center (random intercept). In addition, the difference of patient distribution over different categories of bleeding duration between LV perforation compared to MDV perforation was tested using chi-squared tests.

We visualized the survival probability by bleeding duration and type of perforated vessel using Kaplan-Meier curves and assessed for differences in survival for three predictor variables (i.e. minimum and maximum bleeding duration, and perforation site) using Cox proportional hazards model, adjusted for age and NIHSS at admission.

Statistical analysis was performed by a professional statistical analyst (N.R.) using R v4.3.2 (https://www.r-project.org/). *p*-Values ⩽0.05 were deemed significant and no adjustment for multiple testing was done. This article follows the STROBE reporting guidelines (http://www.strobe-statement.org).

## Results

Screening of 43.364 thrombectomies performed in 34 centers in North America, Europe and Australia yielded 461 cases of vessel perforation. Two patients were excluded from final analysis: One did not show definite extravasation on angiography. In the second, vessel perforation led to a direct carotid-cavernous fistula but not to subarachnoid hemorrhage. The final analysis comprised 459 patients (mean age 72.5 ± 13.6 years, 270 (58.8%) female; patient baseline characteristics: see Table 1).

**Table 1.** Patient baseline characteristics including sites of initial occlusion and sites of perforation.

Baseline characteristics	All patients (*n* = 459) (%)
Age (years, mean ± SD)	72.5 ± 13.6
Gender (female patients)	270 (58.8)
Pre-stroke mRS (*n* = 450)	
0	270 (60.0)
1	76 (16.9)
2	49 (10.9)
3	42 (9.3)
4	13 (2.9)
NIHSS at admission (mean ± standard deviation)	14 ± 7.5
Occlusion site^a^	
Intracranial internal carotid artery	63 (13.7)
Vertebral artery	7 (1.5)
Basilar artery	39 (8.5)
M1	161 (35.1)
M2	180 (39.2)
M3	27 (5.9)
M4	4 (0.9)
A1	8 (1.7)
A2	12 (2.6)
A3	8 (1.7)
P1	11 (2.4)
P2	2 (0.4)
P3	1 (0.2)
Perforation site	
*LV perforation*	155 (33.8)
Intracranial internal carotid artery	26 (5.7)
Vertebral artery	5 (1.1)
Basilar artery	16 (3.5)
M1	69 (15.0)
M2	39 (8.5)
*MDV perforation*	296 (64.5)
M2	146 (31.8)
M3	90 (19.6)
M4	12 (2.6)
ACOM	1 (0.2)
A1	4 (0.9)
A2	4 (0.9)
A3	4 (0.9)
A4	3 (0.7)
PCOM	2 (0.4)
P1	24 (5.2)
P2	2 (0.4)
P3	2 (0.4)
PICA	1 (0.2)
AICA	1 (0.2)
*Small vessel/perforator branch perforation*	8 (1.7)
Lenticulostriatal perforators	4 (0.9)
Thalamic perforators	3 (0.7)
Anterior choroid artery	1 (0.2)

mRS: Modified Rankin Scale; NIHSS: National Institutes of Health Stroke Scale; LV: large vessel; MDV: medium distal vessel; M1–M4, A1–A4, and P1–P3: corresponding segments of middle, anterior and posterior cerebral artery; ACOM: anterior communicating artery; PCOM: posterior communicating artery; PICA: posterior inferior cerebellar artery; AICA: anterior inferior cerebellar artery.

a 53 Patients presented with more than one intracranial occlusion.

NIHSS at admission was 14 ± 7.5. Intravenous thrombolysis was administered in 191 patients (41.4%). Thrombectomy was performed to treat LVO in 267 patients (58.2%) and MDVO in 192 patients (41.8%, sites of initial vessel occlusion: see Table 1). The majority of the interventionalists in charge were neuroradiologists or radiologists within neuroradiology subspecialization training (*n* = 187, 81%). The remaining interventionalists were neurosurgeons (*N* = 17, 7%), general interventional radiologists (*n* = 16, 7%) and neurologists (*n* = 10, 4%). Thrombectomy was performed in general anesthesia in 240 patients (52.3%), in conscious sedation in 178 patients (38.8%), in local anesthesia in 40 patients (8.7%) and without anesthesia in 1 patient (0.2%).

### Outcome at 90 days

Overall mortality at 90 days was 51.9% and 16.3% of patients reached good functional outcome at 90 days. The following factors were independently associated with worse mRS at 90 days: Worse pre-stroke mRS, unsuccessful recanalization at thrombectomy, higher NIHSS at admission, longer time from onset to admission and LV perforation as compared to MDV perforation (details see Supplemental Table S1).

### Effect of thrombolysis

**Figure 1.**
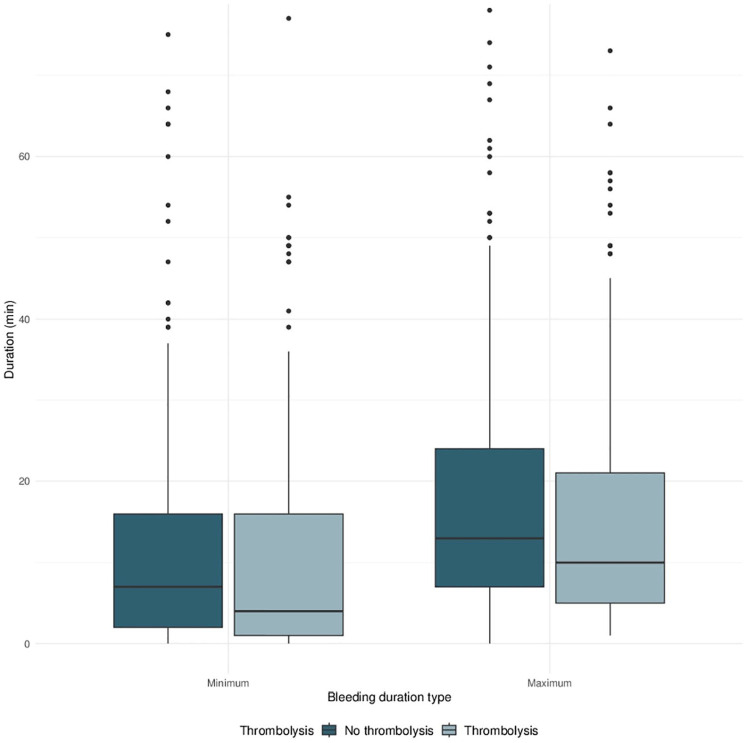
Box plots of bleeding duration for patients under thrombolysis versus patients without thrombolysis. Left: Box plots for minimum bleeding duration. Right: Box plots for maximum bleeding duration.

In proportional odds model analysis, no significant independent association between thrombolysis administration and mRS at 90 days was found (adjusted odds ratio (aOR) 0.655, *p* = 0.054, details see Supplemental Table S1). Patients who received thrombolysis did not show longer bleeding duration compared to patients who did not receive thrombolysis (*p* = 0.98 and *p* = 0.30 for minimum and maximum bleeding duration, see Figure 1 and Supplemental Material). Medication aiming to restore or to enhance coagulation was used in 19 patients having received thrombolysis (10%, details see Supplemental Table S4).

### Site of perforation

In 155 patients (33.8%) an LV had been perforated, while perforation occurred at an MDV in 296 patients (64.5%) and in small vessels/perforator branches in 8 patients (1.7%, details see Table 1).

**Figure 2.**
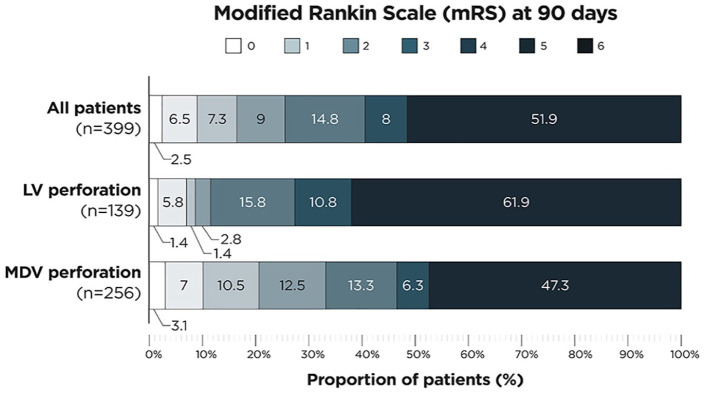
Modified Rankin Scale (mRS) at 90 days of: (1) all patients with vessel perforation during thrombectomy (upper graph), (2) patients with large vessel perforation (LV perforation, middle graph), and (3) patients with medium/distal vessel perforation (MDV perforation, lower graph). Four patients suffering from perforation of small vessels are included in the upper graph, too.

When compared to MDV perforation, LV perforation was independently associated with worse mRS at 90 days (aOR 1.709, *p* = 0.04). Patients with LV perforation reached good functional outcome at 90 days in 8.6% and their 90 days overall mortality was 61.9%, compared to 20.6% good functional outcome and 47.3% mortality in MDV perforation (see Figure 2).

**Figure 3.**
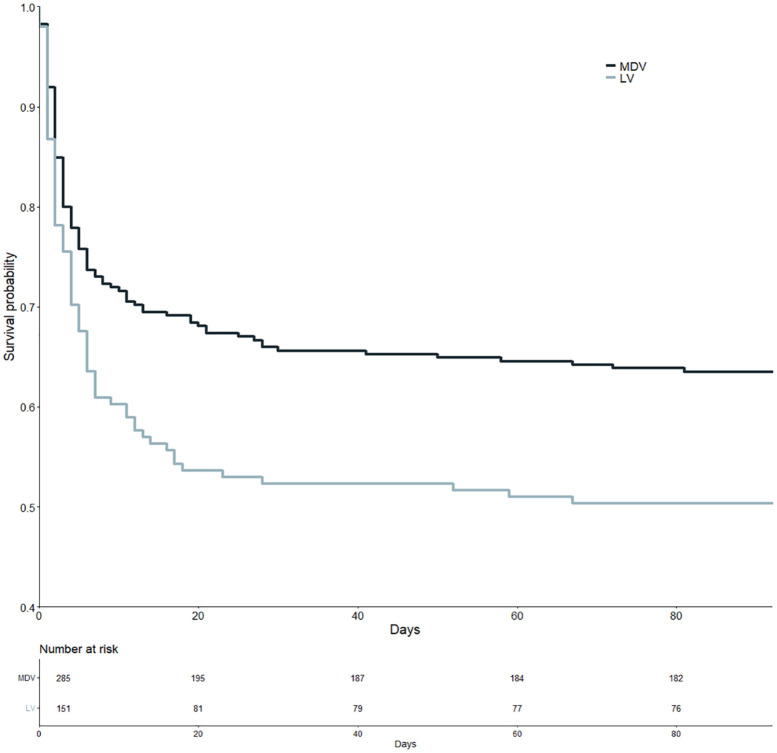
Kaplan-Meier curves of patients with medium/distal vessel (MDV) perforation (dark blue) and patients with large vessel (LV) perforation (light blue-gray).

Survival of patients with LV perforation was significantly worse compared to patients with MDV perforation (hazard ratio (HR) 1.389 and *p* = 0.021, see Figure 3).

There was no significant association between bleeding duration and type of perforated vessel (see Supplemental Material). Patients with LV perforation did not show long bleeding duration significantly more often compared to patients with MDV perforation (*p* = 0.35 and *p* = 0.06 for minimum and maximum bleeding duration, respectively).

### Bleeding duration

Median minimum bleeding duration was 6 min (Interquartile range (IQR) 2–16 min). Median maximum bleeding duration was 11 min (IQR 5–23 min).

**Figure 4.**
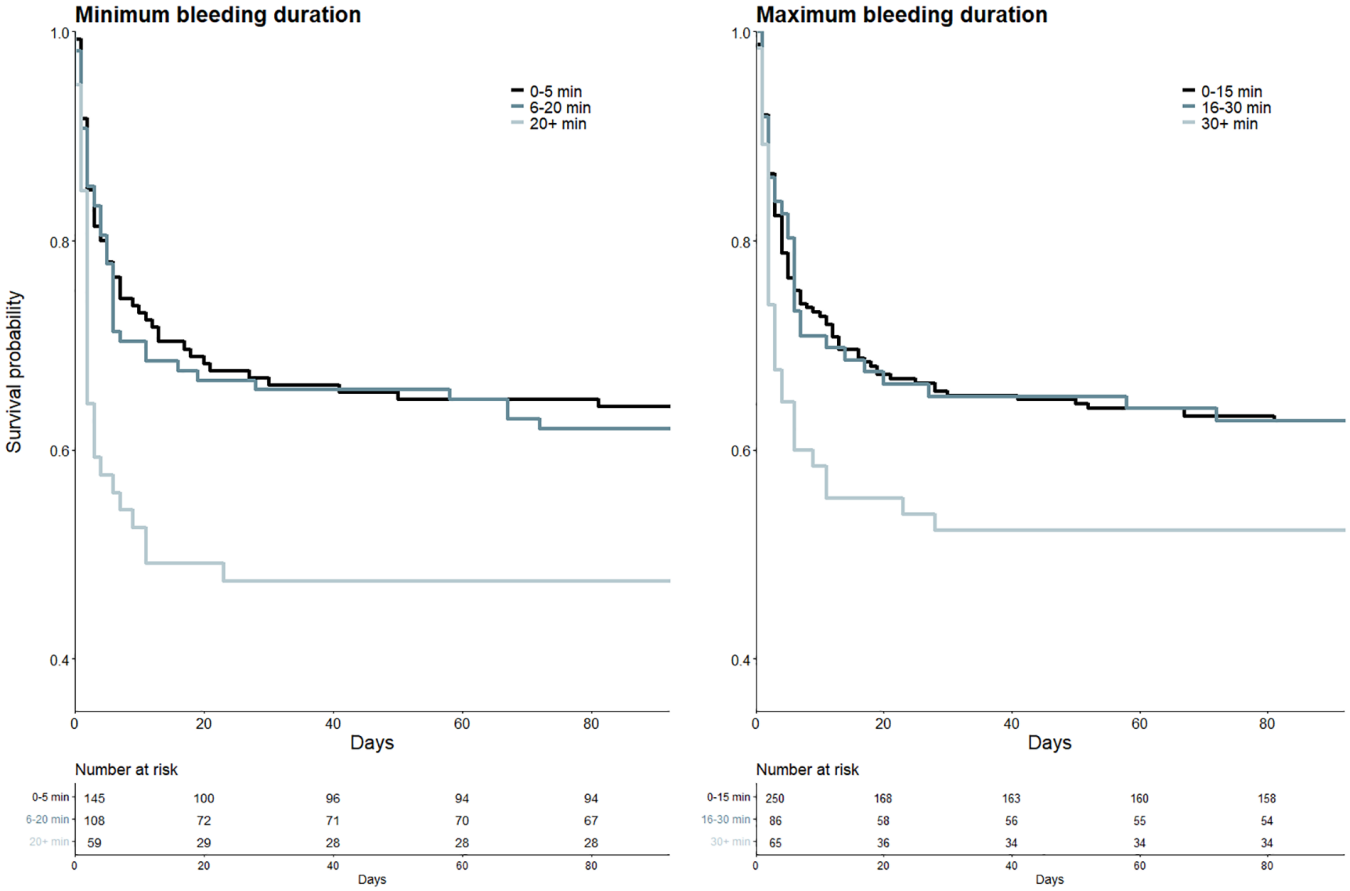
Kaplan-Meier curves of patients with different bleeding durations. Left: Kaplan-Meier curves for patients with minimum bleeding duration of 0–5 min (black), 6–20 min (blue) and >20 min (light blue-gray). Right: Kaplan-Meier curves for patients with maximum bleeding duration of 0–15 min (black), 15–30 min (blue) and >30 min (light blue-gray).

Longer bleeding duration was associated with significantly worse mRS at 90 days (aOR 1.013, *p* = 0.05 and aOR 1.010, *p* = 0.045 for minimum and maximum bleeding duration, respectively). Survival of patients with long bleeding duration was significantly worse (HR 1.797 and *p* = 0.009 for minimum bleeding duration >20 min, HR 1.614 and *p* = 0.013 for maximum bleeding duration >30 min, respectively; see Figure 4). Survival of patients with intermediate bleeding duration was not significantly worse compared to patients with short bleeding duration (HR 1.279 and *p* = 0.201 for minimum and HR 1.151 with *p* = 0.452 for maximum bleeding duration, respectively). Factors associated with longer bleeding duration are presented in the Supplemental Tables S2 and S3.

### Endovascular hemostatic therapy

Endovascular hemostatic treatment was performed in 214 patients (46.6%). The first-line approach was a temporary measure including inflation of balloon guide catheter, inflation of an intracranial balloon or temporary coil placement without detachment in 144 patients. Permanent vessel occlusion was chosen as first-line approach in 70 patients, including permanent coiling, injection of a liquid embolic agent or a combination of both (details see Table 2).

**Table 2.** First-line strategies for endovascular hemostatic therapy and the corresponding time to bleeding cessation (minutes).

Time between initiation of hemostatic ENT and cessation of active bleeding (minutes)					
	Mean	Median	SD	Min	Max
Temporary hemostatic measures (*n* = 144)	14	8	18	0	95
Inflation of balloon guide catheter (*n* = 37)	16	11	19	0	80
Inflation of intracranial balloon (*n* = 69)	16	8	19	0	95
Temporary coil placement without detachment (*n* = 38)	11	5	14	0	71
Permanent vessel occlusion (*n* = 70)	8	4	13	0	65
Permanent coiling only (*n* = 53)	7.4	2	12	0	65
Injection of liquid embolic only (*n* = 12)	4.5	4	4.2	0	12
Coiling - liquid embolic (*n* = 5)	23	12	21	6	51

ENT: endovascular treatment.

In the subgroup who received endovascular hemostatic treatment, bleeding duration was significantly longer compared to patients without endovascular hemostatic treatment (median difference 6 min; *p* < 0.001 and median difference 5 min, *p* < 0.001 for minimum and maximum bleeding duration, respectively). Neurointerventional experience of the interventionalist was higher in the subgroup of patients who received endovascular treatment of the perforation (median difference 2 years, p = 0.001). A significant difference was found between temporary hemostatic measures and permanent vessel occlusion regarding the time needed to achieve bleeding cessation (median difference: 4 min, *p* < 0.001, details see Table 2).

### Recanalization

At the time of perforation, successful recanalization (mTICI ⩾ 2b) was achieved in 134 patients (29.8%). The final angiographic result showed successful recanalization in 201 patients (44.3%). In 90 patients (19.7%), endovascular thrombectomy was pursued after the occurrence of the vessel perforation. The mTICI score could be improved by continuation of thrombectomy in 53/90 patients (58.9%). In 14 patients, the mTICI score improved without further thrombectomy maneuvers.

In patients with mTICI < 2b at the time of perforation, mRS at 90 days did not differ significantly between patients in which thrombectomy was pursued after perforation and patients in which thrombectomy was aborted (OR 1.139, *p* = 0.59).

## Discussion

In this large, retrospective, multicenter cohort study, vessel perforation during thrombectomy was associated with poor clinical outcomes and an overall mortality of 51.9%.

Patients who received thrombolysis prior to vessel perforation did not show statistically significant longer bleeding duration or worse outcome at 90 days compared to patients without thrombolysis. This has to be interpreted with caution, since patients under anticoagulation and/or platelet inhibition at admission might not have received thrombolysis but might have bled in a prolonged fashion nonetheless, thereby biasing the comparison. Furthermore, despite accounting for covariates found to be independently associated with the primary outcome, confounding by indication might have biased the results since patients admitted late after onset, with substantial infarcts or very high NIHSS might not have received thrombolysis.

A recent meta-analysis of six large prospective trials has evaluated whether thrombectomy alone is non-inferior compared to thrombectomy with thrombolysis.^22^ Despite analyzing 2313 patients, the number of vessel perforations in this meta-analysis is rather small. Therefore, our data add valuable evidence to the question whether thrombolysis should be performed by shedding light on a patient subcohort for which prospective trials can provide only limited data. Our results are reassuring that even in case of vessel perforation, thrombolysis is not proven to increase patient harm.

In our patient cohort, perforation of a large vessel was associated with worse mRS at 90 days and lower survival probability when compared to medium/distal vessel perforation. Higher flow rates of extravasation in large vessel perforation may contribute to this effect. Furthermore, if permanent vessel occlusion is deemed necessary, patients with permanent occlusion of a large vessel may develop larger infarcts compared to patients with permanent occlusion of a medium/distal vessel. In their cohort of 74 patients, van der Sluijs et al.^17^ found a similar trend, which however was not significant in adjusted regression analysis, possibly owing to the smaller number of patients in their study.

A wide range of bleeding duration was reported. In patients in whom the minimum and maximum bleeding duration exceeded 20 and 30 min, respectively, worse functional outcome and lower survival probability were observed. This should encourage interventionalists to initiate hemostatic measures as rapidly as possible with the aim of stopping active bleeding within 20 min.

Patients receiving endovascular hemostatic therapy had longer bleeding duration. This indicates probably that patients with rapid spontaneous bleeding cessation were not considered for hemostatic therapy. A quicker cessation of active bleeding was achieved if the first-line hemostatic strategy was permanent vessel occlusion as opposed to temporary hemostatic measures and experienced interventionalists used hemostatic measures more often than their less experienced colleagues. This underlines that physicians performing revascularizing interventions should be trained in complication management including the use of intracranial balloons, coils, and liquid embolic agents.

Overall, successful recanalization (mTICI ⩾ 2b) was associated with better mRS at 90 days. However, in patients with unsuccessful recanalization at the time of perforation, continuation of thrombectomy was not associated with better mRS at 90 days compared to abortion. The likelihood of good clinical outcome is reported to decrease with an increasing number of retrieval attempts,^23^ which may contribute to this finding. In their case series of patients with vessel perforation during thrombectomy, Ducroux et al.^15^ reported an association between favorable outcome and successful reperfusion. However, they did not limit their analysis to patients with initial unsuccessful recanalization at the time of perforation. From the perspective of an interventionalist who needs to decide whether thrombectomy should be pursued after the event of vessel perforation, an analysis including patients who already achieved successful recanalization at this stage cannot be directly used to inform decision making.

This study has several limitations: the retrospective, non-randomized design limits comparison between patient groups, for example regarding thrombolysis. The study cohort consists exclusively of patients with periprocedural vessel perforation which prohibits comparison to patient cohorts without perforation, for example, regarding interventionalists’ experience.

This study’s strengths include the high number of included patients and the international multicenter design which increase the generalizability of our results and might limit the possible selection bias. The statistical approach with center-adjusted analysis meets the requirements given by the clustered structure of the data, thereby further reducing bias.

## Conclusion

Vessel perforation during thrombectomy is a severe complication which is strongly linked to poor functional outcome and death. This retrospective cohort study does not suggest that thrombolysis is associated with worse outcome if perforation occurs. Associations with worse functional outcome or death were found for longer bleeding duration and perforation of a large vessel, emphasizing the need for rapid bleeding control. Both temporary and permanent endovascular hemostatic strategies can be effective, but permanent vessel occlusion results in quicker bleeding cessation. These data do not suggest that thrombolysis should be withheld because of the possibility of periprocedural vessel perforation.

## Supplemental Material

sj-docx-1-eso-10.1177_23969873241272542 – Supplemental material for “Insights into vessel perforations during thrombectomy: Characteristics of a severe complication and the effect of thrombolysis” Supplemental material, sj-docx-1-eso-10.1177_23969873241272542 for “Insights into vessel perforations during thrombectomy: Characteristics of a severe complication and the effect of thrombolysis” by Victor Schulze-Zachau, Nikki Rommers, Nikolaos Ntoulias, Alex Brehm, Nadja Krug, Ioannis Tsogkas, Matthias Mutke, Thilo Rusche, Amedeo Cervo, Claudia Rollo, Markus Möhlenbruch, Jessica Jesser, Kornelia Kreiser, Katharina Althaus, Manuel Requena, Marc Rodrigo-Gisbert, Tomas Dobrocky, Bettina L Serrallach, Christian H Nolte, Christoph Riegler, Jawed Nawabi, Errikos Maslias, Patrik Michel, Guillaume Saliou, Nathan Manning, Alexander McQuinn, Alon Taylor, Christoph J Maurer, Ansgar Berlis, Daniel PO Kaiser, Ani Cuberi, Manuel Moreu, Alfonso López-Frías, Carlos Pérez-García, Riitta Rautio, Ylikotila Pauli, Nicola Limbucci, Leonardo Renieri, Isabel Fragata, Tania Rodriguez-Ares, Jan S Kirschke, Julian Schwarting, Sami Al Kasab, Alejandro M Spiotta, Ahmad Abu Qdais, Adam A Dmytriw, Robert W Regenhardt, Aman B Patel, Vitor Mendes Pereira, Nicole M Cancelliere, Carsten Schmeel, Franziska Dorn, Malte Sauer, Grzegorz M Karwacki, Jane Khalife, Ajith J Thomas, Hamza A Shaikh, Christian Commodaro, Marco Pileggi, Roland Schwab, Flavio Bellante, Anne Dusart, Jeremy Hofmeister, Paolo Machi, Edgar A Samaniego, Diego J Ojeda, Robert M Starke, Ahmed Abdelsalam, Frans van den Bergh, Sylvie De Raedt, Maxim Bester, Fabian Flottmann, Daniel Weiss, Marius Kaschner, Peter T Kan, Gautam Edhayan, Michael R Levitt, Spencer L Raub, Mira Katan, Urs Fischer and Marios-Nikos Psychogios in European Stroke Journal

sj-docx-2-eso-10.1177_23969873241272542 – Supplemental material for “Insights into vessel perforations during thrombectomy: Characteristics of a severe complication and the effect of thrombolysis” Supplemental material, sj-docx-2-eso-10.1177_23969873241272542 for “Insights into vessel perforations during thrombectomy: Characteristics of a severe complication and the effect of thrombolysis” by Victor Schulze-Zachau, Nikki Rommers, Nikolaos Ntoulias, Alex Brehm, Nadja Krug, Ioannis Tsogkas, Matthias Mutke, Thilo Rusche, Amedeo Cervo, Claudia Rollo, Markus Möhlenbruch, Jessica Jesser, Kornelia Kreiser, Katharina Althaus, Manuel Requena, Marc Rodrigo-Gisbert, Tomas Dobrocky, Bettina L Serrallach, Christian H Nolte, Christoph Riegler, Jawed Nawabi, Errikos Maslias, Patrik Michel, Guillaume Saliou, Nathan Manning, Alexander McQuinn, Alon Taylor, Christoph J Maurer, Ansgar Berlis, Daniel PO Kaiser, Ani Cuberi, Manuel Moreu, Alfonso López-Frías, Carlos Pérez-García, Riitta Rautio, Ylikotila Pauli, Nicola Limbucci, Leonardo Renieri, Isabel Fragata, Tania Rodriguez-Ares, Jan S Kirschke, Julian Schwarting, Sami Al Kasab, Alejandro M Spiotta, Ahmad Abu Qdais, Adam A Dmytriw, Robert W Regenhardt, Aman B Patel, Vitor Mendes Pereira, Nicole M Cancelliere, Carsten Schmeel, Franziska Dorn, Malte Sauer, Grzegorz M Karwacki, Jane Khalife, Ajith J Thomas, Hamza A Shaikh, Christian Commodaro, Marco Pileggi, Roland Schwab, Flavio Bellante, Anne Dusart, Jeremy Hofmeister, Paolo Machi, Edgar A Samaniego, Diego J Ojeda, Robert M Starke, Ahmed Abdelsalam, Frans van den Bergh, Sylvie De Raedt, Maxim Bester, Fabian Flottmann, Daniel Weiss, Marius Kaschner, Peter T Kan, Gautam Edhayan, Michael R Levitt, Spencer L Raub, Mira Katan, Urs Fischer and Marios-Nikos Psychogios in European Stroke Journal
