# Correction to: Insights into vessel perforations during thrombectomy: Characteristics of a severe complication and the effect of thrombolysis

**DOI:** 10.1093/esj/aakag109

**Published:** 2026-09-01

This is a correction to: Victor Schulze-Zachau, Nikki Rommers, Nikolaos Ntoulias, Alex Brehm, Nadja Krug, Ioannis Tsogkas, Matthias Mutke, Thilo Rusche, Amedeo Cervo, Claudia Rollo, Markus Möhlenbruch, Jessica Jesser, Kornelia Kreiser, Katharina Althaus, Manuel Requena, Marc Rodrigo-Gisbert, Tomas Dobrocky, Bettina L Serrallach, Christian H Nolte, Christoph Riegler, Jawed Nawabi, Errikos Maslias, Patrik Michel, Guillaume Saliou, Nathan Manning, Alexander McQuinn, Alon Taylor, Christoph J Maurer, Ansgar Berlis, Daniel PO Kaiser, Ani Cuberi, Manuel Moreu, Alfonso López-Frías, Carlos Pérez-García, Riitta Rautio, Ylikotila Pauli, Nicola Limbucci, Leonardo Renieri, Isabel Fragata, Tania Rodriguez-Ares, Jan S Kirschke, Julian Schwarting, Sami Al Kasab, Alejandro M Spiotta, Ahmad Abu Qdais, Adam A Dmytriw, Robert W Regenhardt, Aman B Patel, Vitor Mendes Pereira, Nicole M Cancelliere, Carsten Schmeel, Franziska Dorn, Malte Sauer, Grzegorz M Karwacki, Jane Khalife, Ajith J Thomas, Hamza A Shaikh, Christian Commodaro, Marco Pileggi, Roland Schwab, Flavio Bellante, Anne Dusart, Jeremy Hofmeister, Paolo Machi, Edgar A Samaniego, Diego J Ojeda, Robert M Starke, Ahmed Abdelsalam, Frans van den Bergh, Sylvie De Raedt, Maxim Bester, Fabian Flottmann, Daniel Weiss, Marius Kaschner, Peter T Kan, Gautam Edhayan, Michael R Levitt, Spencer L Raub, Mira Katan, Urs Fischer, Marios-Nikos Psychogios, Insights into vessel perforations during thrombectomy: Characteristics of a severe complication and the effect of thrombolysis, *European Stroke Journal*, Volume 10, Issue 1, 1 March 2025, Pages 63–73, https://doi.org/10.1177/23969873241272542

In the originally published version of this manuscript, the thirty-sixth co-author’s name was incorrectly transposed.

This should read: “Pauli Ylikotila”.

This correction has been made only in this correction notice to preserve the version of record.

