# Supplementary material for: Insights into vessel perforations during thrombectomy: Characteristics of a severe complication and the effect of thrombolysis
Source: Eur Stroke J. 2024 Aug 22;10(1):63–73. doi: 10.1177/23969873241272542 (PMC11569593; doi:10.1177/23969873241272542)
Supplement: sj-docx-1-eso-10.1177_23969873241272542 – Supplemental material for “Insights into vessel perforations during thrombectomy: Characteristics of a severe complication and the effect of thrombolysis” [file sj-docx-1-eso-10.1177_23969873241272542.docx]

**Supplemental Material**

|  | **aOR** | **CI** | **p-value** |
| --- | --- | --- | --- |
| **Thrombolysis vs. no thrombolysis** | 0.655 | [0.425, 1.008] | 0.054 |
| **Initial LVO vs. MDVO** | 1.464 | [0.920, 2.331] | 0.108 |
| **Pre-stroke mRS (compared to pre-stroke mRS 0)** |  |  |  |
| mRS 1 | 1.938 | [1.094, 3.436] | 0.023* |
| mRS 2 | 2.994 | [1.359, 6.579] | 0.007* |
| mRS 3 | 1.988 | [0.984, 4.016] | 0.056 |
| mRS 4 | 5.291 | [1.058, 26.316] | 0.042* |
| **Higher NIHSS on admission (per NIHSS point)** | 1.056 | [1.027, 1.087] | < 0.01* |
| **Unsuccessful recanalization (mTICI<2b) vs. successful recanalization** | 1.969 | [1.286, 3.014] | 0.002* |
| **Time from onset to admission (6-24h vs. 0-6h)** | 1.712 | [1.016, 2.890] | 0.044* |
| **LV perforation vs. MDV perforation** | 1.709 | [1.031, 2.833] | 0.038* |

**Supplemental Table S1:** Results of proportional odds model regarding independent association with higher mRS at 90 days.

aOR: Adjusted odds ratio. CI: confidence interval. LVO: large vessel occlusion. MDVO: medium / distal vessel occlusion. mRS: modified Rankin Scale. NIHSS: National Institute of Health Stroke Scale. mTICI: Modified treatment in cerebral infarction score. LV: large vessel. MDV: medium / distal vessel.

|  | Estimate | CI | p-value |
| --- | --- | --- | --- |
| LVO vs. MDVO | 0.661 | [-3.752, 5.105] | 0.77 |
| Procedural step: retraction of stentretriever / aspiration catheter vs. navigation beyond occlusion | -0.448 | [-6.184, 5.104] | 0.875 |
| LV perforation vs. MDV perforation | 2.869 | [-1.774, 7.495] | 0.226 |
| Relative aspiration catheter size (per mm) | -1.176 | [-4.186, 1.833] | 0.445 |
| Relative stent retriever size (per mm) | 0.01 | [-2.470, 2.518] | 0.994 |
| Thrombolysis vs. no thrombolysis | -0.059 | [-4.380, 4.252] | 0.978 |

**Supplemental Table S2:** Effect of different factors on minimum bleeding duration in univariate analyses.

CI: confidence interval. LVO: large vessel occlusion. MDVO: medium /distal vessel occlusion. LV: large vessel. MDV: medium / distal vessel.

|  | Estimate | CI | p-value |
| --- | --- | --- | --- |
| LVO vs. MDVO | 3.092 | [-1.225, 7.444] | 0.162 |
| Procedural step: retraction of stentretriever / aspiration catheter vs. navigation beyond occlusion | 1.416 | [-3.986, 6.734] | 0.603 |
| LV perforation vs. MDV perforation | 3.783 | [-0.790, 8.353] | 0.106 |
| Relative aspiration catheter size (per mm) | -3.708 | [-6.792, -0.634] | 0.02 |
| Relative stent retriever size (per mm) | 1.88 | [-0.928, 4.901] | 0.235 |
| Thrombolysis vs. no thrombolysis | -2.288 | [-6.641, 1.997] | 0.297 |

**Supplemental Table S3:** Effect of different factors on maximum bleeding duration in univariate analyses.

CI: confidence interval. LVO: large vessel occlusion. MDVO: medium /distal vessel occlusion. LV: large vessel. MDV: medium / distal vessel.

| **Medication** | **Number of patients** |
| --- | --- |
| Platelet transfusion | 4 |
| Fibrinogen | 3 |
| Tranexamic acid | 3 |
| Prothrombin complex concentrate | 3 |
| Factor Eight Inhibitor Bypassing Agent (FEIBA) | 2 |
| Cryoprecipitate | 2 |
| Andexanet Alfa | 1 |
| Vitamin K | 1 |
| Not further specified | 2 |

**Supplemental Table S4:** Usage of medications after perforation occurrence to restore / enhance coagulation.
